# Association between Body Mass Index and Self Body Image Perception

**Published:** 2017-12

**Authors:** Cristiana Lucretia POP

**Affiliations:** Dept. of Physical Education and Sports, Faculty of Agrifood and Environmental Economics, The Bucharest University of Economic Studies, Bucharest, Romania

## Dear Editor-in-Chief

The research reflects the difference between BMI values and the weight perception and explains how young women tend to distort their physical dimensions. The body image disturbed perception can be quantified in around 55% of subjects with normal weight, but perceiving their self-body as being mostly too heavy and less frequently too thin. Distorted self-perception of body image is often associated with insecure attitude, preoccupation or seeking reassurance in peer’s opinion. Body image perception, as a subjective component of well-being, is significantly correlated with the anthropometrical measurements, which are relative stable objective data. Overall, 41% (r^2^=0.41) of body dissatisfaction is a consequence of weight and fat deposits determining a greater BMI value. Body image can be enhanced in a percentage of 41% with physical activities; as is known, the most commonly mentioned PE goals are the improvement of health state and a harmonious physical development. Even body image acceptance is a subjective issue, depending on cultural and social factors, yet we find a consistent, statistical significant correlation with BMI (r (158) = 0.64, *P*<0.0005). It is reasonable to consider BMI values as useful predictors of body dissatisfaction risk among young female.

A percentage of 79% of the young women in this study sample reported dissatisfaction with their body image. The study was undertaken in 2016 in Bucharest University of Economic Studies on a sample of female students attending physical education classes. The mean value of body dissatisfaction index in our sample is BDI = −1.2 considering that in average each young women reported an ideal thinner shape and a slander figure comparing with the actual perceived silhouette.

The present findings seem to be consistent with other studies that found various values of body dissatisfaction in young women samples from different cultures. The range of these scores varied from 47.3 % in Brazil (1), 65.6% in Poland (2); 73.6% in Saudi Arabia (3) up to 100% in Pakistan (4). “This data confirm the thesis that landmarks that society promotes are very severe for most girls and young women and put them in a position of inferiority, repercussions on self-esteem and self-confidence” (5).

**Fig. 1:**
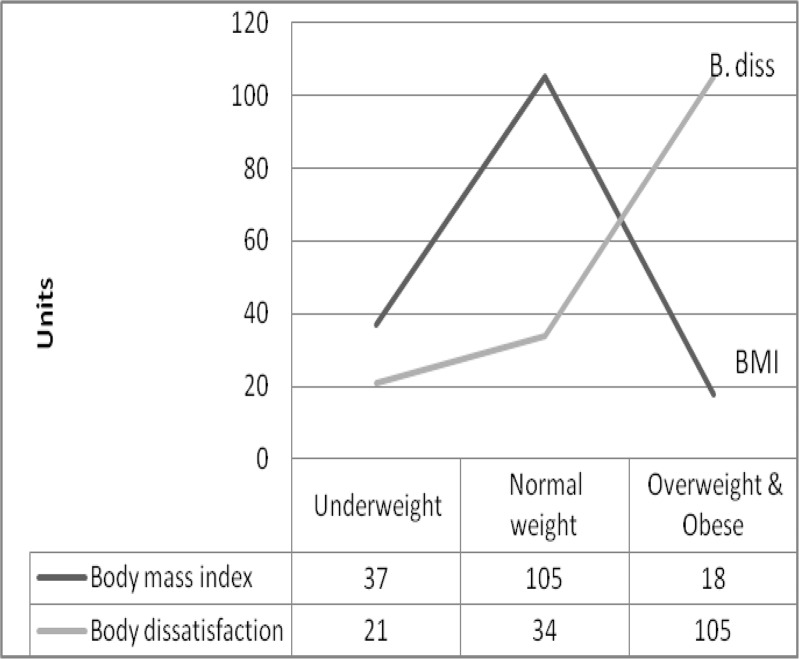
Body dissatisfaction index versus BMI distribution

This study discloses the unbreakable relationship between mind and body, meaning that every intervention at physical level will have effects on emotional well-being too. This holistic approach is a valuable educational mean in promoting mentalities, attitudes and behaviors favorable to a healthy and active lifestyle (5). The findings of the current study indicate that the majority of young women perceive their physical appearance as being too heavy, even the BMI values indicate a prevalence of underweight and normal weight women in the studied sample. “Under the social and cultural patterns pressure a majority of women are not satisfied with their body shape regardless the BMI values and identifies their ideal body with a thinner version” (5). Even underweight persons are willing to lose weight in order to correspond to an expected physical attractiveness standard (Fig. 1).

A possible and plausible explanation for this eternal and quasi-globalized constant preoccupation for losing weight is the body composition. A low percentage of muscle mass, with high density and an increased percentage of body fat, greater in volume could result in a normal BMI, but in a disharmonic body shape.

Women dissatisfaction with physical appearance or body parts and their over adaptation to an ultrathin beauty ideal could lead to diet and emotional disorders. Physical and health education can enhance individuals understanding and acceptance of their own and others corporeality, and provide the cognitive constructs for setting and assuming realistic goals.
